# Excitotoxicity: Still Hammering the Ischemic Brain in 2020

**DOI:** 10.3389/fnins.2020.579953

**Published:** 2020-10-26

**Authors:** Dennis W. Choi

**Affiliations:** Department of Neurology, SUNY Stony Brook, Stony Brook, NY, United States

**Keywords:** excitatory amino acids, glutamate, zinc, stroke, hypoxia, ischemia, excitotoxicity, neurotoxicity

## Abstract

Interest in excitotoxicity expanded following its implication in the pathogenesis of ischemic brain injury in the 1980s, but waned subsequent to the failure of N-methyl-D-aspartate (NMDA) antagonists in high profile clinical stroke trials. Nonetheless there has been steady progress in elucidating underlying mechanisms. This review will outline the historical path to current understandings of excitotoxicity in the ischemic brain, and suggest that this knowledge should be leveraged now to develop neuroprotective treatments for stroke.

## Introduction

**FIGURE 1:**
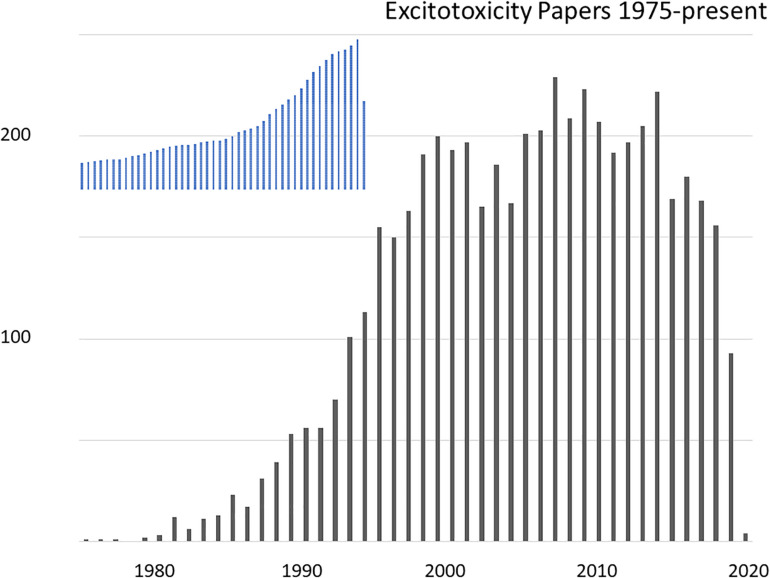
Research articles on the topic of excitotoxicity indexed in PubMed, by year 1975-present. The search was for articles indexed under a MeSH term of “acid, excitatory amino”, containing “excitotoxicity” OR “neurotoxicity” in any field. For shape comparison, the inset show total indexed papers over the same period with a peak in 2019 of 1.39 million papers.

It has now been 63 years since Lucas and Newhouse (1957) discovered the ability of parenterally administered glutamate to kill central neurons in the rodent retina, and 51 years since Olney (1969) extended this observation to neurons in the hypothalamus and hippocampus. Subsequently finding that a series of structurally related neuroexcitatory amino acids exhibited similar neurotoxicity, with potencies corresponding to known neuroexcitant potencies, Olney et al. (1974) proposed that the “necrotizing effect is, in essence, an exaggeration of the excitatory effect” and coined the term “excitotoxic amino acids”. These seminal observations remained relatively fallow until the 1980s, when advances in excitatory amino acid (EAA) pharmacology led to widespread recognition of glutamate’s neurotransmitter function and scientific interest in EAAs burgeoned. Over the next two decades, the subfield of excitotoxicity likewise expanded, driven most prominently by its implication in the pathogenesis of ischemic brain damage, and several pharmaceutical companies developed glutamate receptor antagonists as candidate treatments for stroke. By the early 2000s these drugs had failed in clinical trials and excitotoxicity research lost substantial momentum. A reflection of the excitotoxicity research boom and subsequent slowdown can be found in the number of relevant publications indexed by PubMed (Figure 1).

However, implication of excitotoxicity as an agent of human diseases has not gone away. The current issue of *Frontiers in Neuroscience* is timely, marking a half-century of active excitotoxicity research. I thank the editors for my invitation to contribute, and will provide here a brief, semi-chronological, and at times personal, overview of this research, focusing on the still extant path forward for developing an anti-excitotoxic neuroprotective treatment for stroke. Recent reviews on the topic have appeared (Wu and Tymianski, 2018; Fern and Matute, 2019; Hardingham, 2019; Ge et al., 2020). Other articles in this issue will likely discuss the possible contribution of excitotoxicity to neurodegeneration in chronic diseases such as amyotrophic lateral sclerosis or Alzheimer’s disease.

## Excitotoxicity – Early Days (Up to Early 1990s)

Brain tissue contains high concentrations of free glutamate, in the 5–15 mmol/kg range (Schousboe, 1981), but until the 1970s this glutamate was widely assumed to be serving purely metabolic functions. Hayashi (1954) speculated that glutamate might be a neurotransmitter based on convulsant activity on motor cortex, an idea that was strongly supported by direct demonstration of neuroexcitatory properties (Curtis et al., 1960). Subsequent research on the topic of glutamate signaling proceeded gently for nearly two decades – a lull Watkins and Jane (2009) have dubbed “the dark ages”. But evidence for synaptic synthesis, Ca^2+^-dependent release and rapid cellular uptake accrued (Fagg and Lane, 1979), and pharmacological tools were developed that enabled critical testing of the transmitter hypothesis. Glutamate receptors were classified into N-methyl-D-aspartate (NMDA) and non-NMDA types, the latter subsequently further divided into kainite and quisqualate receptors (Watkins and Evans, 1981), and then later, kainate, α-amino-3-methyl-4-isoxazoleproprionic acid (AMPA), and G protein-coupled metabotropic receptors (mGluRs) (Krogsgaard-Larsen et al., 1980; Sugiyama et al., 1987; Hollmann and Heinemann, 1994); molecular subunit nomenclature is still evolving (Collingridge et al., 2009). Glutamate antagonists blocked endogenous neural signaling in multiple pathways, identifying glutamate as a neurotransmitter at insect and crustacean neuromuscular junctions, and then throughout the mammalian CNS (Nistri and Constanti, 1979; Cotman et al., 1981; McLennan, 1983).

Olney’s initial considerations of how excitotoxicity might contribute to human disease focused on the possible dangers of exogenous agonists, especially the monosodium glutamate added to food as a flavor enhancer (Olney, 1982). Recognition of ubiquitous glutamate neurotransmission elevated interest in the possible pathophysiological importance of endogenous stores, and excitotoxicity gained further attention as a laboratory tool, useful for creating “axon-sparing” lesions in brain. These threads came together when injection of kainate into the rat striatum was found to reproduce some of the anatomical and biochemical features of Huntington’s disease, raising speculation that progressive excitotoxic damage might contribute to its pathogenesis (Coyle and Schwarcz, 1976; Mcgeer and Mcgeer, 1976). Further, the convulsant properties of kainate and the dependence of kainate neurotoxicity *in vivo* upon intact glutamatergic afferents supported a role for excitotoxicity in epileptic brain injury (Ben-Ari et al., 1980; Meldrum, 1985). Thus, the stage was well set for three breakthrough studies that leveraged newly available receptor antagonists to implicate endogenous excitotoxicity in ischemic brain damage.

1. Rothman (1984) showed that the glutamate antagonist γ-D-glutamylglycine (DGG) could block glutamate-induced depolarization and cell swelling, as well as anoxic injury in cultured rat hippocampal neurons. This key study followed up on his earlier demonstration that 10 mM MgCl_2_ could protect the neurons against damage induced by cyanide exposure (Rothman, 1983).
2. Simon R. et al. (1984) found that direct hippocampal injection of the selective NMDA antagonist, 2-amino-7-phosphonoheptanoic acid (APH), reduced pathological changes in nearby neurons 2 h after transient global ischemia (TGI) in rats. In retrospect, the study assessed neuronal morphology too soon to determine lasting survival, as it was just becoming recognized that selective neuronal death after TGI can occur days later (Kirino, 1982). Regardless, it demonstrated a neuroprotective effect of NMDA receptor (NMDAR) blockade against a component of ischemic injury *in vivo*.
3. Wieloch (1985) injected APH into the rat caudate, and observed markedly improved survival of nearby neurons 1 week after 30 m exposure to hypoglycemia, further implicating NMDARs in acute brain injury.

Additionally, microdialysis measurements revealed that brain ischemia induced within minutes a large increase in extracellular glutamate emanating from depolarized nerve terminals and astrocytes (Benveniste et al., 1984), indicating that neurons in the ischemic brain would inevitably be exposed to elevated glutamate concentrations.

It is worth pausing here. The entire cell death field was then a shadow of what it would become. (PubMed papers retrieved by “cell death” and published in 1985 are about 2% of the 14,000 such papers published in 2019). Few people considered either acute or chronic neurodegeneration a worthwhile research topic – cells die, right – and the neurologists of the day managed patients presenting with Alzheimer’s disease or stroke nihilistically, attending mainly to accompanying medical conditions. Early excitotoxicity studies truly changed “normal science” (Kuhn, 1962), suggesting that some ischemic neuronal cell death occurred consequential to specific events and pathways accessible to therapeutic interdiction.

Inspired by Rothman’s *in vitro* studies, I set out to investigate glutamate neurotoxicity in the mouse cortical cell culture system I had going at the time. Having worked during the 1970s as a graduate student on γ-aminobutyric acid (GABA) signaling and benzodiazepines, I was emerging from clinical training as a neurologist and setting up my own laboratory at Stanford, newly funded to study the electrophysiology of glutamate receptors. Shifting research focus on the fly was entirely feasible – thank you, NIH.

I found that bath exposure to 500 μM glutamate caused cultured cortical neurons to swell immediately and then proceed to disintegrate, similar to the “intracellular edema and neuronal necrosis” observed by Olney (1969) in mouse brains after glutamate injection. However, I wondered if leaving glutamate in the bath, as Rothman had done, might not exaggerate cell swelling and attendant damage over what would occur *in vivo* within a three-dimensional brain and closed skull. I decided therefore to terminate the bath exposure after 5 m, an exposure still widely lethal by the next day. Replacing extracellular Na^+^ with an impermeant cation eliminated acute neuronal swelling, but most neurons still went on to degenerate over the next hours. In contrast, removing extracellular Ca^2+^ increased early cell swelling, and yet most cells recovered and survived (Choi, 1985). This and other experiments (Choi, 1987; Choi et al., 1987) suggested that glutamate neurotoxicity at lower exposure levels was predominantly driven by delayed, Ca^2+^ -dependent processes rather than by the immediate entry of Na^+^ responsible for neuroexcitation and, together with Cl^–^ and water, immediate excitotoxic swelling.

A Ca^2+^ -dependent death fit with observations of EAA-induced Ca^2+^ movement into brain tissue (Heinemann and Pumain, 1980; Berdichevsky et al., 1983), and aligned excitotoxic death with a larger theme of Ca^2+^ overload in other types of cell death, including the toxin-induced death of hepatocytes (Schanne et al., 1979) and agonist- or mechanical injury-induced muscle cell death (Bloom and Davis, 1972; Fleckenstein et al., 1975; Leonard and Salpeter, 1979). It also meshed with prior implications of Ca^2+^ overload in the neuronal death induced by ischemia (Siesjö, 1981; Simon R.P. et al., 1984) or prolonged seizures (Griffiths et al., 1982). Measurements with newly available Ca^2+^-sensitive microelectrodes revealed a rapid and large drop in brain extracellular Ca^2+^ after the onset of ischemia (Harris et al., 1981). The toxic EC_50_ for glutamate with 5 m exposure was 50–100 μM in mixed astrocyte + neuron cultures (Choi et al., 1987), dropping to 5 μM with 30 m exposure in astrocyte-poor cultures lacking protective cellular uptake (Rosenberg et al., 1992).

The exciting discovery that NMDA but not kainate gated Ca^2+^-permeable channels (MacDermott et al., 1986) suggested that NMDARs would play a foreground role in glutamate neurotoxicity. This was borne out. The selective NMDA antagonist 2-amino-5-phophonovalerate (APV) only modestly reduced the neuroexcitation or acute neuronal cell swelling induced by brief glutamate exposure, but markedly reduced later cell death (Choi et al., 1988). Selective NMDAR block was also effective in reducing the neuronal death induced by hypoxia (Weiss et al., 1986; Goldberg et al., 1987), glucose deprivation (Monyer et al., 1989) or mechanical trauma (Tecoma et al., 1989).

Non-NMDA agonists were also potently neurotoxic on cortical neurons, but prolonged exposures of several hours was required to produce widespread cell death. Consistent with the effects of glutamate + APV, kainate induced immediate excitotoxic neuronal swelling, but if exposure was terminated at 5 m, most cells recovered and survived (Koh et al., 1990). We considered it likely that this more slowly triggered neurotoxicity mediated by kainate or AMPA receptors (KARs, AMPARs) was mediated by slower Ca^2+^ overload secondary to excessive Na^+^ entry, involving voltage-gated Ca^2+^ channels and reverse operation of the electrogenic Na^+^/Ca^2+^ exchanger, NCX (Choi, 1988). In that mode, favored under conditions of membrane depolarization, high internal [Na^+^] and low external [Na^+^], NCXs provide high capacity transport for 3 Na^+^ ions out coupled to 1 Ca^2+^ ion in. The importance of NCXs to excitotoxicity was supported by implication in anoxic optic nerve injury (Stys et al., 1992). Later we showed that net cellular ^45^Ca^2+^ accumulation induced by NMDA or glutamate was much larger/faster than that induced by high K^+^, kainate, or AMPA (Hartley et al., 1993), and Hyrc et al. (1997) found a similar relationship for [Ca^2+^]_*i*_.

**FIGURE 2:**
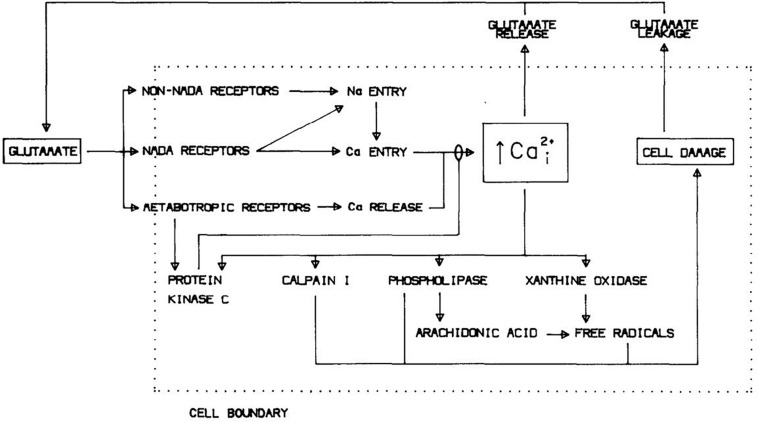
Mechanisms underlying glutamate neurotoxicity as of 1988. Reprinted from Choi (1988).

Identification of excessive Ca^2+^ entry as the primary mediator of excitotoxicity, likely augmented by Ca^2+^ release from endoplasmic reticulum (ER, triggered by mGluR activation; see below), also bridged to prior studies implicating free radical generation in the pathogenesis of ischemic brain injury (Siesjö, 1981). Multiple Ca^2+^-dependent enzymes including calpains, endonucleases and lipases, were known to be capable of damaging cells, and Ca^2+^ overload would impair mitochondrial energy production and perturb Ca^2+^-dependent signaling pathways (Cheung et al., 1986; Orrenius et al., 1989). But the generation of free radicals seemed especially well positioned to drive lethal cytodegeneration. Consequent to cellular Ca^2+^ overload, breakdown of lipid membranes into fatty acids mediated by phospholipase A_2_ and further metabolism via prostaglandin and leukotriene pathways, damage to mitochondrial electron transport and conversion of xanthine dehydrogenase to xanthine oxidase all generate free radicals, promoting lipid peroxidation and membrane failure (Chan et al., 1985; Traystman et al., 1991; Halliwell, 1992). Testing the hypothesis, 21-aminosteroid lipid peroxidation inhibitors attenuated both glutamate neurotoxicity and oxygen-glucose deprivation (OGD)-induced neuronal death (Monyer et al., 1990). Toxic NMDA exposure increased superoxide formation in cultured cerebellar neurons, and trapping this superoxide was neuroprotective (Lafon-Cazal et al., 1993). Subsequent studies used electron paramagnetic resonance spectroscopy and oxidation state-sensitive fluorescent dyes to detect mitochondrial production of reactive oxygen species (ROS) after NMDAR-mediated Ca^2+^ overload, and demonstrated that this production could be substantially reduced by inhibition of mitochondrial electron transport or dissipation of the mitochondrial membrane potential (Dugan et al., 1995; Reynolds and Hastings, 1995). Figure 2 shows a diagram of excitotoxicity mechanisms as we saw it in 1988.

The discovery that NMDAR-mediated Ca^2+^ influx activated neuronal nitric oxide synthase (NOS) suggested that nitric oxide (NO) might be both a normal neuronal signaling molecule and a free radical mediator of glutamate neurotoxicity (Garthwaite et al., 1988; Bredt et al., 1990). Freely diffusible through membranes, NO released from neurons activates soluble guanylate cyclase in neighboring cells including smooth muscle. Cytotoxicity at higher concentrations was harnessed by macrophages to kill target cells, with NO reacting with superoxide to form the highly reactive nitrogen species (RNS), peroxynitrite, and promoting destructive hydroxyl radical formation (Halliwell and Gutteridge, 1984; Beckman et al., 1990). Pivotal experiments were carried out by Dawson et al. (1991), demonstrating that inhibition of neuronal NOS (nNOS) selectively attenuated NMDAR-mediated neurotoxicity but not kainate neurotoxicity. Later, they would show that a major toxic consequence of NMDA-induced NO formation was the hyperactivation of poly(ADP-ribose) polymerase (PARP), likely consequential to peroxynitrite-induced DNA damage and resulting in cellular energy depletion and nuclear translocation of apoptosis-inducing factor (AIF), triggering a caspase-independent death (“parthanatos”). Subsequent studies from other labs would raise a possible role for oxidative intracellular Zn^2+^ release in connecting NO to PARP overactivation (see below).

As a sidebar, inhibiting nNOS did not reduce glutamate neurotoxicity in the cortical cultures my lab was using in the 1990s. We came to regard this as an idiosyncrasy, likely reflecting low baseline nNOS expression and/or a large astrocyte presence sopping up NO. Raising inducible NOS (iNOS) expression in the astrocyte layer did add a NOS-dependent component to NMDAR-mediated toxicity in the cultures (Hewett et al., 1994). In any case, this culture system difference illustrates that more than one potentially lethal downstream cascade can be triggered in parallel by glutamate receptor overactivation and robustly kill neurons. A messy “**and**”, not an “**or**”: a race to death. Whether a specific cascade ends up directly responsible for cell death such that its inhibition improves survival, and the exact features of that death, may depend on where that cascade lies in an injury hierarchy, as well as on the identity and closeness of competitive injury pathways that may leave behind their own incomplete molecular and morphological signatures.

## NMDA Antagonists, Stroke, and Apoptosis

Demonstration of the neuroprotective effects of glutamate antagonists against hypoxia/ischemia/hypoglycemia *in vivo* and *in vitro*, together with identification of NMDAR overactivation and Ca^2+^ overload as primary mediators of acute glutamate toxicity, encouraged global efforts to test NMDA antagonist drugs in various animal models of brain ischemia and develop drugs suitable for human use. The first drug candidate out of the gate was Merck’s MK-801, a potent and selective NMDA antagonist with excellent brain penetrance. Gill et al. (1987) reported that pretreatment with MK-801 reduced neuronal death in gerbil brains subjected to either bilateral or unilateral forebrain ischemia. Benefits of NMDA antagonists later proved variable in models of TGI, in some cases perhaps confounded by hypothermia (Buchan and Pulsinelli, 1990), but reduction of infarction or disability was robustly observed with NMDA antagonists given up to 2 h after focal brain ischemia in rodents or larger animals (Gotti et al., 1988; Kochhar et al., 1988; Ozyurt et al., 1988; Park et al., 1988; Steinberg et al., 1988). By 1992 close to 20 published studies reported this finding. AMPA/kainate antagonists also were neuroprotective in brain ischemia studies, especially after TGI (Sheardown et al., 1990). In 1994, Huang et al. reported that mice lacking nNOS exhibited reduced infarcts after permanent occlusion of the middle cerebral artery (pMCAO) (Huang et al., 1994).

However, the NMDA antagonist drug candidates brought into development by several companies for use in stroke all failed in clinical testing. Some were abandoned because of side effects; others lacked efficacy. In two cases (CNS-1102, CGS 19755), there were worrisome trends toward worse outcome in the treated group (Hoyte et al., 2004; Ginsberg, 2008; Lai et al., 2014). As those reviewers noted, clinical studies did not match up well with the earlier animal studies. Some drug dosing was too low (limited by mechanism-associated behavioral side effects such as hallucinations) or too late (most dosing was > 3 h post stroke onset, and in some trials up to 48 h post onset, as enrolling patients at earlier time points was not practical at that time). A smaller number of pilot studies with AMPA antagonists were also disappointing, in one case (ZK20075) unsurprisingly depressing consciousness (Ginsberg, 2008).

The first wave of NMDA antagonist stroke trials was followed by two significant improvements, but these second-generation efforts also failed. The first improvement, informed by insights into the molecular biology and subunit composition of glutamate receptors, was the use of antagonists selective for NR2B/GluN2B. The predominant expression of NR2B in forebrain vs. cerebellum and limbic areas raised hopes of achieving neuroprotection with less dose-limiting side effects. Furthermore, as discussed below, consideration of Ca^2+^ source specificity and NMDAR signaling relationships suggested that NR2B contributes more to excitotoxic death than the other major forebrain subtype, NR2A. A older antagonist, ifenprodil, was discovered to be conveniently NR2B selective, attractively use-dependent and neuroprotective in multiple animal models of global and focal brain ischemia (Wang and Shuaib, 2005; Gogas, 2006). New molecular entity congeners of ifenprodil, SL-82.0715 and CP-101,606 were developed by Synthélabo and Pfizer, respectively, and exhibited reduced side effects compared to pan NMDAR antagonists. But the former failed to show efficacy in a stroke trial and the latter was felled by electrocardiographic toxicity (QT prolongation) after showing some promise against severe traumatic brain injury (Yurkewicz et al., 2005; Lai et al., 2014).

The second improvement was in stroke trial methodology. Recognizing that treatment delay was problematic in prior stroke neuroprotection trials, Saver et al. (2015) completed a remarkable multicenter study (FAST-MAG) in which intravenous magnesium sulfate was given to 1700 acute stroke patients by paramedics within 2 h (and often within 1 h) of stroke onset. Still no improvement in outcome measures was observed. An earlier study of Mg^2+^ treatment for stroke (IMAGES) with a 12 h treatment window was also negative (Muir et al., 2004). While the methodological advance represented by FAST-MAG is clear, direct relevance of FAST-MAG and IMAGES to the hypothesis of NMDAR-mediated excitotoxicity in human stroke is less so. Mg^2+^ has multiple actions that might contribute to its modest neuroprotective effects in animal stroke models, and it is likely that its block of NMDA channels in the ischemic brain would be substantially relieved by cellular depolarization (Mayer et al., 1984; Nowak et al., 1984).

Enthusiasm for developing anti-excitotoxic therapies for stroke was also progressively dampened by the lack of efficacy in stroke trials demonstrated by several other drugs targeting related mechanisms, including voltage-gated Ca^2+^ channels (nimodipine), free radicals (tirilazad mesylate, ebselen, NXY-059), phospholipid hydrolysis (citicoline), and nitric oxide synthase (lubeluzole). These trials also had shortcomings in concept or design, commonly involving the use of doses and delay time windows that were not supported by the enabling preclinical studies (Ginsberg, 2008; Sutherland et al., 2012). Administration of a lipophilic Ca^2+^/Zn^2+^ chelator, DP-b99, within 9 h of stroke onset also failed in Phase III testing (Lees et al., 2013).

The free radical drugs tested would be considered less than compelling candidates today in updated comparisons. Neither tirilazad (Hall, 1997) nor NXY-059 (Kuroda et al., 1999) penetrate well into brain parenchyma, and NYX-059 exhibited little antioxidant activity on cultured neurons (Antonic et al., 2018). Ebselen is a mimic of glutathione peroxidase that would be expected to decompose only a subset of harmful radicals (hydroperoxides); it has little aqueous solubility and requires a thiol co-substrate like glutathione to maintain activity. A fourth antioxidant drug, edaravone, has a favorable chemical profile, being amphiphilic and able to directly scavenge a range of harmful lipid- and water-soluble radicals (Watanabe et al., 2018). It has protective effects in multiple animal models of brain ischemia, and showed sufficient evidence of neuroprotective efficacy in clinical testing to gain approval for use in stroke in Japan (Lapchak, 2010). Available published data documented a just significant (*p* = 0.048) beneficial effect on 90 d clinical outcome in patients treated within 72 h of stroke onset; clear benefit was suggested in an exploratory analysis including only patients treated within 24 h (Edaravone Acute Infarction Study Group, 2003). Subsequent clinical trials demonstrating ability to slow the progression of amyotrophic lateral sclerosis supported the premise of human neuroprotective activity, and led to US FDA approval in 2017 for that indication (Radicava) (Cruz, 2018).

By 2001, I had moved to work in the pharmaceutical industry and was in a position to champion an improved NMDA antagonist/stroke trial, but I had become worried that there was potentially a conceptual problem with hard blocking NMDARs in the ischemic brain: apoptosis (Choi, 1995; Lee et al., 1999; Ikonomidou and Turski, 2002; Papadia and Hardingham, 2007). Programmed cell death had come to center stage in biological research, spearheaded by elegant genetic studies in *C. elegans* (Ellis and Horvitz, 1986; Hengartner and Horvitz, 1994) and growing appreciation that apoptosis occurs in a wide range of disease states outside of normal development. Hypoxic-ischemic cell death was long considered to be a defining example of a non-apoptotic, “accidental” death – necrosis – triggered by “violent and non-physiological” environmental changes (Wyllie et al., 1980). It, like classic excitotoxicity, was associated with prominent cellular and organellar swelling and membrane rupture, in contrast to the controlled cellular condensation characteristic of apoptosis. Yet evidence was steadily emerging suggesting that some neurons underwent apoptosis after ischemia, in particular selectively vulnerable neurons dying in delayed fashion after TGI (Goto et al., 1990; Shigeno et al., 1990); but also after focal ischemia (Linnik et al., 1993; MacManus et al., 1994), especially threshold insults triggering “very delayed infarction” days later (Du et al., 1996; Endres et al., 1998).

As expected, intense excitotoxic death appeared typically not to be apoptotic (Ignatowicz et al., 1991; Dessi et al., 1993; Choi, 1996). However, slowly triggered AMPAR/KAR-mediated neuronal death was associated with internucleosomal DNA cleavage, consistent with incomplete activation of apoptosis pathways (Gwag et al., 1997), and neuronal apoptosis occurred after mild excitotoxic insults, either in young cultures with limited EAA receptor expression (Kure et al., 1991) or with lower concentrations of NMDA (Bonfoco et al., 1995). These studies fit with findings that apoptosis could be induced by Ca^2+^ overload (Wyllie et al., 1984) or oxidative stress (Lennon et al., 1991), including NO (Albina et al., 1993). More recent studies have indicated that Ca^2+^ and oxidative surges can interact at the ER-mitochondrial signaling interface to trigger mitochondrial membrane permeabilization, permitting cytochrome c and AIF release, and the activation of caspase-dependent or caspase-independent apoptosis pathways (Tajeddine, 2016; Hempel and Trebak, 2017; Humeau et al., 2018). Or resulting in necrosis, if mitochondrial and cellular failure is fulminant.

Together, these observations suggested that excitotoxicity, like many other insults, has the potential to trigger regulated cell death programs; but when intense, membrane, energy and protein synthesis failure destroy neurons before these programs can complete. Ca^2+^ ionophores likewise can induce neuronal apoptosis at low concentrations, and necrosis at high concentrations (Gwag et al., 1999). A death race hierarchy was already apparent in cultured cortical neurons deprived of oxygen and glucose: blockade of rapidly triggered NMDAR-mediated death was necessary to reveal AMPAR/KAR-dependent death. Blocking both NMDAR and AMPAR/KAR-mediated neurotoxicity rendered neurons resistant to prolonged OGD, but then a further increase in OGD duration (“blocked OGD”) drove neurons into apoptosis (Gwag et al., 1995; Choi, 1996).

Consideration of ischemic apoptosis suggested that a sustained high degree of NMDAR blockade could be harmful. While presumably beneficial initially in reducing acute excitotoxic necrosis and the triggering of ischemic apoptosis, sustained blockade risks keeping [Ca^2+^]_*i*_ below a necessary survival “set-point” (Koike et al., 1989). U-shaped curves are ubiquitous in cell and organismal biology, with deleterious effects induced by both too little and too much of many metabolites, messengers, drugs, etc. Apoptosis of young sympathetic neurons induced by nerve growth factor (NGF) withdrawal can be attenuated by raising [Ca^2+^]_*i*_ from a basal level of 90 nM to about 240 nM, the same higher basal level found in older neurons capable of surviving without NGF (Koike and Tanaka, 1991). This survival-promoting level of [Ca^2+^]_*i*_ is considerably lower than the levels associated with excitotoxic exposure to glutamate, which can exceed 10 μM (Stout and Reynolds, 1999) and are associated with much larger amounts of net cellular calcium loading than that induced by survival-promoting activation of voltage-gated Ca^2+^ channels (Hartley et al., 1993). Lowering extracellular Ca^2+^ or reducing membrane Ca^2+^ channel opening induces apoptosis in a wide range of cell types [reviewed in Canzoniero et al. (2004)]. Many signaling pathways transduce the ability of moderate Ca^2+^ levels to promote cell survival, including the activation of PI3K/Akt/mTOR, Ras/Raf/ERK, and AMPK pathways, as well as modulation of gene expression by CREB and NFAT family transcription factors downstream of Ca^2+^ / calmodulin (Pinto et al., 2015; Feldmann et al., 2018; Varghese et al., 2019).

**FIGURE 3:**
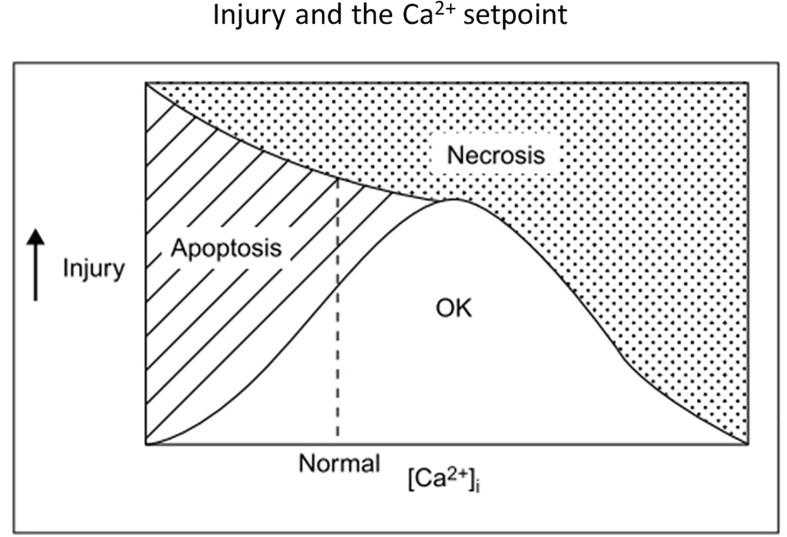
Old but possibly still useful concept diagram illustrating relationships among insult severity, net [Ca^2+^]_*i*_ and the survival-apoptosis-necrosis continuum. A single insult might lead either to apoptosis or necrosis, depending on insult severity and [Ca^2+^]_*i*_, with low [Ca^2+^]_*i*_ or milder insults favoring apoptosis. Optimal Ca^2+^ setpoints may also apply to neurite outgrowth and nerve growth cone movements (Mattson and Kater, 1987). Reprinted from Choi (1995).

Supporting the premise of Ca^2+^ starvation in neurons undergoing ischemic apoptosis, [Ca^2+^]_*i*_ was abnormally low in neurons dying after exposure to blocked OGD, and normalizing [Ca^2+^]_*i*_ with the voltage-gated Ca^2+^ channel opener, S)-(-)-BayK-8644, or even low concentrations of kainate improved neuronal survival (Canzoniero et al., 2004). 1–2 days NMDAR blockade alone reduced cultured neuronal [Ca^2+^]_*i*_ below baseline levels and triggered / enhanced apoptosis (Takadera et al., 1999; Snider et al., 2002; Yoon et al., 2003); injection of a single dose of MK-801 induced neuronal apoptosis within hours in the developing rat brain (Ikonomidou et al., 1999). Consistent with a benefit of low level glutamate receptor activation *in vivo*, delayed administration of the partial NMDA agonist, D-cycloserine, improved functional outcome in rats after traumatic brain injury (Adeleye et al., 2010) or focal ischemia (Dhawan et al., 2011). Figure 3 illustrates in broad brush strokes how neuronal [Ca^2+^]_*i*_, apoptosis and necrosis might interrelate after excitotoxic / ischemic insults.

In sum, whether NMDA antagonists are beneficial or harmful in the ischemic brain may depend on multiple insult and treatment specifics, with key issues being the relative proportion of necrosis vs. apoptosis taking place; and the timing, location and amount of [Ca^2+^]_*i*_ lowering induced by the drugs. A dynamic that might slow excitotoxic necrosis and thus increase the overall contribution of apoptosis to ischemic brain damage is a reduction of extracellular Na^+^ and Ca^2+^, as documented with ion-sensitive electrodes (Hansen, 1985); also see below under Potassium). Furthermore, ischemic apoptosis might be expected to be more prominent in human stroke, with its sometimes stuttering onset and potentially large penumbral areas across complex gyral anatomy, than after surgical artery occlusion in some lissencephalic rat stroke models. Another reason why early subtype-unspecific NMDA antagonists might have disappointed in human stroke, despite their routine effectiveness in rat stroke.

## Expansion and Refinement of Excitotoxicity Concepts

### NMDARs and Ca^2+^ Source Specificity

Returning to the excitotoxicity story, the next major advance in the 1990s was appreciating that Ca^2+^ source makes a difference. Noting that elevations of [Ca^2+^]_*i*_ mediated by voltage-gated Ca^2+^ channels lacked the toxicity associated with NMDAR activation, Tymianski et al. (1993) proposed that NMDARs were preferentially linked to downstream mediators of excitotoxic injury. The discovery that the PDZ domains of both NR2B (Moon et al., 1994) and nNOS (Brenman et al., 1996) interacted with PDZ domains of the postsynaptic density protein, PSD-95, fit the bill, and furthermore placed NR2B in the foreground of excitotoxicity. Suppressing PSD-95 protein expression in cultured neurons attenuated both NMDA-induced NO production and neuronal death, without affecting NMDA currents or NMDA-induced Ca^2+^ loading (Sattler et al., 1999).

Extension of the Ca^2+^ source specificity hypothesis was pursued in studies assessing the relative contributions of extrasynaptic and synaptic subpopulations of NMDARs to excitotoxic death. Synaptic and extrasynaptic NMDA receptors exhibit similar single channel properties (Clark et al., 1997) but have been reported to trigger different transcription factor and gene expression changes; activation of extrasynaptic but not synaptic NMDARs caused rapid loss of mitochondrial membrane potential and neuronal death (Hardingham et al., 2002; Karpova et al., 2013). Mediation of excessive Ca^2+^ influx into mitochondria was postulated to be favored by extrasynaptic NMDAR activation, possibly due to spatial proximity (Bading, 2017). However, the distinction between synaptic and extrasynaptic receptors may not be sharp, as burst stimulation of inputs to hippocampal CA1 neurons activates both populations (Harris and Pettit, 2008). And arguing against synaptic localization *per se* altering NMDAR contributions to excitotoxicity, delocalizing synaptic NMDARs by depolymerizing F-actin did not alter NMDAR-induced current, Ca^2+^ loading, or cell death. The delocalization did attenuate death after OGD, consistent with synaptic localization increasing exposure to the glutamate released by nerve terminals (Sattler et al., 2000).

Comparing cell death after intense activation of extrasynaptic vs synaptic NMDARs, some investigators have proposed that these subpopulations have opposing effects on excitotoxic death: extrasynaptic receptors promoting death, and synaptic receptors promoting survival (Hardingham et al., 2002; Hardingham, 2009; Lai et al., 2011; Parsons and Raymond, 2014; Wu and Tymianski, 2018). Consistent with that proposal, synaptic activity repressed expression of the mitochondrial Ca^2+^ uniporter, Mcu and reduced neuronal vulnerability to excitotoxic death, a plausible neuroprotective adaptive mechanism (Qiu et al., 2013). However, while supported by careful experiments, the receptor location hypothesis is best considered to be still under test. It does not fit easily with the prominence of PSD-95/nNOS signaling in NMDAR-mediated excitotoxicity, since association with PSD-95 is a hallmark of synaptic localization. Furthermore, as outlined above, the ability of NMDAR activation to reduce apoptosis at lower levels and yet drive excitotoxic apoptosis or necrosis at higher levels can be explained in terms of net Ca^2+^ fluxes and Ca^2+^ setpoints, without invoking this additional level of source specificity. The experimental necessity of utilizing different paradigms to activate synaptic vs extrasynaptic receptors (eg, bicuculline + 4-aminopyridine to stimulate the former, bath glutamate + prior MK-801 to stimulate the latter) leaves open the possibility that outcome differences primarily reflect differences in ionic current envelopes or even just overall Ca^2+^ influx, rather than differences in the fundamental properties of extrasynaptic vs. synaptic receptors. The equivalence of cellular Ca^2+^ loading achieved by the extrasynaptic vs. synaptic activation paradigms is not assured by measuring peak [Ca^2+^]_*i*_, especially if high affinity indicators like Fluo-3 are used, as these may saturate below micromolar excitotoxic elevations (Hyrc et al., 1997; Stout and Reynolds, 1999). Additionally, modulatory influences present differentially at synapses vs elsewhere, e.g., pH changes or Zn^2+^ released by nerve terminals (see below), or differential levels of cell injury may affect outcomes. The receptor location hypothesis might not matter much in stroke anyway, as a large buildup of extracellular glutamate would probably overstimulate both synaptic and extrasynaptic receptors.

In the strongest formulation of Ca^2+^ source specificity, NMDAR subtypes have been assigned both to distinct locations and opposing roles, with NR2A assigned to synaptic locations and pro-survival roles, and NR2B assigned to extrasynaptic locations and pro-death roles (Liu et al., 2007). However, electrophysiological examination of ifenprodil sensitivity in cultured hippocampal neurons suggested that NR2A and NR2B can both be located in either synaptic or extrasynaptic compartments (Thomas et al., 2006). This formulation will also need adjustment to accommodate other subunit compositions of NMDARs, as many are likely triheteromeric, containing both NR2A and NR2B subunits (Tovar et al., 2013).

Additional to activation of nNOS, other NR2B-linked signaling pathways have been proposed to be mediators of excitotoxic death, including death-associated protein kinase 1 (DAPK1) (Nair et al., 2013), phosphatase and tensin homolog deleted on chromosome 10 (PTEN) (Ning et al., 2004) and NOX2 (see below). DAPK1 was recruited to the NR2B protein complex after transient middle cerebral artery occlusion (tMCAO), binding to a unique region of the NR2B C-terminal region, phosphorylating NR2B and upregulating channel current; administration of a peptide uncoupling DAPK1 from NR2B or genetic deletion of DAPK1 reduced infarction after tMCAO and selective neuronal death after TGI (Tu et al., 2010). However, another study did not find evidence of DAPK1 modifying NR2B after excitotoxic insults, or reduced neuronal death after TGI in *Dapk1*^–/–^ mice (McQueen et al., 2017). Downregulating PTEN expression with antisense oligodeoxynucleotides enhanced Akt signaling and reduced the death of vulnerable hippocampal CA1 neurons after TGI (Zhang et al., 2007).

Likely many signaling pathways affecting cell death are triggered by glutamate receptor overactivation, not only via direct signaling linkages, but also unspecifically consequent to cellular damage. Example of the latter are ROS/RNS-induced DNA single strand breaks activating PARP, or mitochondrial damage releasing ROS and cytochrome c. Another example of an unspecific link would be damage to ER and accumulation of misfolded proteins, activating the PERK/eIF2 pathway, increasing levels of ATF4 and CHOP, and promoting apoptosis (Sokka et al., 2007; Almanza et al., 2019). Furthermore, whether a given downstream signaling pathway or event is ultimately responsible for excitotoxic death, or even whether it promotes death vs survival, may depend on quantitative specifics and cellular state. As discussed above, raising [Ca^2+^]_*i*_ can be either survival-promoting or death-promoting, depending. PI3K/Akt signaling is often survival promoting, but in some settings can enhance apoptosis (Los et al., 2009), and cytoplasmic PI3K activation enhances superoxide production by NOX2 (see below). JNK signaling is usually pro-apoptotic but anti-apoptotic functions have been described (Liu and Lin, 2005); and *Jnk3* ^–/–^ mice exhibited reduced kainate-induced and ischemic apoptosis (Yang et al., 1997; Kuan et al., 2003). Yet another example of context-dependent pathway effects on cell survival would be upregulation of BDNF expression by glutamate receptor-induced Ca^2+^ influx or ischemia (Lindvall et al., 1992; Zafra et al., 1992; Favaron et al., 1993; Hardingham et al., 2002). While BDNF is classically anti-apoptotic, it accentuated excitotoxic neuronal necrosis (Koh et al., 1995) and glutathione depletion-induced death of immature neurons (Ratan et al., 1996).

### NADPH Oxidase

NADPH oxidase (NOX) comprises a family of 7 membrane-active protein complexes, NOX1 through NOX5, DUOX1, and DUOX2, that catalyze the transfer of electrons from NADPH to molecular oxygen, primarily generating superoxide (Bedard and Krause, 2007). While long recognized as a key component of neutrophil antimicrobial defenses, NOX was later found in many tissues, including brain, and implicated in acute and chronic neurodegeneration (Infanger et al., 2006; Sorce and Krause, 2009). NOX1, NOX2 and NOX4 are the main brain isoforms, and mice with genetic deletion of the gp91^*phox*^ catalytic unit of NOX2 exhibited reduced brain infarction after tMCAO, even if neutrophil NOX2 was rescued with a bone marrow transplant (Walder et al., 1997; Tang et al., 2011). Subsequent studies showed that ischemia raised the expression of NOX2 and NOX4 in multiple brain cell types over ensuing hours-days, and that amelioration of ischemic infarction could also be achieved by genetic disruption of NOX4 (Radermacher et al., 2013; Ma et al., 2017). Increased expression of endothelial and neuronal NOX4, which is constitutively active, likely contributes to blood-brain barrier (BBB) breakdown and neuronal death, respectively (Casas et al., 2017).

Brennan et al. (2009) identified NOX2 as a specific source of ROS production after NMDAR overactivation, demonstrating that both NMDA-induced ROS production and neuronal death in cortical cultures were blocked by the NOX2 inhibitor, apocynin, the hexose monophosphate pathway inhibitor, 6-aminonicotinamide (reducing NADPH), or deletion of the NOX2 subunit gene, *p47*^*phox*^, as well as by removal of extracellular Ca^2+^ or inhibition of PKCζ, the latter responsible for phosphorylating and activating p47^*phox*^ (Heinecke et al., 1990; Bedard and Krause, 2007). Subsequent study implicated NR2B and phophoinositide-3 kinase (PI3K) upstream of PKCζ (Brennan-Minnella et al., 2013). PI3K activation is likely mediated by Ca^2+^ / calmodulin (Joyal et al., 1997), as well as possibly through the NR2B C-terminal / PSD-95 signalosome (Chen et al., 2015; Minnella et al., 2018). Another way this signalosome might facilitate NOX2 activation is via Src and Panx1 channel opening; loss of Zn^2+^ homeostasis may also contribute (see below). Importantly, the NR2B/NOX2 pathway is strongly inhibited by small drops in intracellular pH (Lam et al., 2013), so its contribution to ischemic injury may be limited to onset, the outer penumbra and reperfusion (see Protons, below).

### AMPA Receptors

As noted above, AMPA receptors (AMPARs) mediate Na^+^ influx and hence can contribute to excitotoxic Ca^2+^ overload and neuronal death. Although these receptors lack direct linkage to NOS/NOX, they participate substantially in brain damage after focal and global ischemia, in the latter setting typically contributing more than NMDARs to the delayed death of selectively vulnerable neurons (Sheardown et al., 1990; Buchan et al., 1991; Hicks et al., 1999). This prominent contribution to delayed selective neuronal death may be largely due to upregulated expression of Ca^2+^-permeable AMPA receptors.

The Ca^2+^ (and Zn^2+^, see below) permeability of central AMPARs during development and in response to synaptic activity is regulated by the expression of an RNA-edited form of AMPA subunit GluR2 that contains an arginine residue instead of the genetically-coded glutamine in a key position within the second transmembrane domain (Burnashev et al., 1992; Liu and Zukin, 2007). While most central AMPARs contain such edited GluR2 subunits and exhibit low Ca^2+^ permeability, certain telencephalic and cerebellar neurons, including aspiny interneurons and neurons with high levels of nNOS, express Ca^2+^ -permeable AMPARs and are highly vulnerable to AMPA-induced neuronal death (Hope et al., 1991; Weiss et al., 1994). Ischemia enhances the expression of Ca^2+^ -permeable AMPARs in vulnerable neuronal populations (Pellegrini-Giampietro et al., 1992), which then constitute a dominant route for toxic Ca^2+^ /Zn^2+^ entry, limiting the protective reach of NMDA antagonists. This enhancement is mediated by turning on expression of the gene silencer REST, reducing GluR2 promoter activity (Calderone et al., 2003).

### Kainate Receptors

While less studied than other glutamate receptor due to a historical paucity of selective pharmacological tools, kainate receptors (KARs) are expressed postsynaptically at glutamatergic synapses and presynaptically on glutamatergic and GABAergic nerve terminals, modifying transmitter release (Jane et al., 2009; Traynelis et al., 2010). Postsynaptic KARs contribute to synaptic excitation, more at some synapses, such as mossy fiber inputs to hippocampal CA3 neurons, than others; and likewise participate variably in synaptic plasticity. Analogous to AMPARs, a minority of KARs containing unedited GluK1 or GluK2 subunits can be Ca^2+^ permeable and convey heightened vulnerability to glutamate excitotoxicity where heavily expressed, for example on somatostatin-containing interneurons (Sun et al., 2009). Administration of the GluK1R antagonist, LY377770, 2 h after endothelin-1-induced focal ischemia in rats reduced infarction and produced a surprisingly large reduction in extracellular glutamate levels in ipsilateral striatum (O’Neill et al., 2000).

### Metabotropic Glutamate Receptors

Glutamate released from nerve terminals and astrocytes will activate G-protein coupled mGluRs on neurons and glia concurrently with ionotropic glutamate receptors. There are 8 major mGluRs and several splice variants, divided into 3 groups based on structure, function, and pharmacology: Group I (mGluR1, mGluR5) linked to activation of phospholipase C (PLC); and Groups II (mGluR2, mGluR3) and III (mGluR4, mGluR6, mGluR7, mGluR8) linked to inhibition of adenylate cyclase (Schoepp et al., 1990; Pin and Duvoisin, 1995). mGluRs modulate synaptic transmission and plasticity; although they probably do not play a primary role in mediating acute excitotoxic brain injury, they influence this injury and are thus worth keeping in view for possible secondary therapeutic targeting in stroke (Sun et al., 2019).

Group I mGluR-activated PLC generates inositol 1,4,5-trisphosphate, triggering Ca^2+^ release from ER, and diacylglycerol, which together through PKC can enhance excitotoxic Ca^2+^ entry through NMDARs, NCXs and NHEs (see below) (Benquet et al., 2002; Lipp and Reither, 2011); and activate phospholipase A2, promoting ROS formation and lipid peroxidation (Bonventre, 1997; van Rossum and Patterson, 2009; Guemez-Gamboa et al., 2011). Possibly through PLC / diacylglycerol signaling, mGluR1 also opens plasmalemmal TRP (TRPC3) cation channels (see below) (Kim et al., 2003; Hartmann et al., 2011). As expected from these actions, and the typically net excitatory effects of Group I agonists on neural circuits, activation of Group I mGluRs enhanced NMDA-induced neuronal death; and inhibition, especially of mGluR1, reduced that death *in vitro* (Bruno et al., 1995b, 1999; Strasser et al., 1998) as well as ischemic injury *in vivo* (Pellegrini-Giampietro et al., 1999; Li et al., 2013). Besides canonically activating PLC, Group I mGluRs could potentially have neuroprotective effects through G-protein mediated downmodulation of NMDARs (Yu et al., 1997; Bertaso et al., 2010) and neuroprotective Akt signaling via Homer, PIKE-L and nuclear PI3K (Rong et al., 2003; Xu et al., 2007). However at least the latter action may be quickly abrogated by calpain (Xu et al., 2007). Surprisingly though, interfering with the C-terminal interactions between mGluR1 and NR2A with cell-permeable peptides was recently reported to attenuate NMDA-induced neuronal death *in vitro* and infarction after tMCAO (Lai et al., 2019).

In contrast, Group II and III mGluRs most often have inhibitory effects on neural circuits. and anti-excitotoxic effects of Group II or Group III agonists have been observed *in vitro* (Bruno et al., 1995a). The Group III agonist ACPT-1 reduced infarction in rats after tMCAO (Domin et al., 2018). Within Group II however, neuronal mGluR2 activation has been proposed to enhance excitotoxicity, perhaps by limiting GABA release (Corti et al., 2007). A novel selective mGluR2 negative allosteric modulator, ADX92639, reduced selective neuronal death after TGI (Motolese et al., 2015).

### Protons

In addition to Na^+^ and Ca^2+^, another cation participating in excitotoxicity is H^+^. Rapid local changes in extracellular pH accompany physiological neuronal activity and modulate the behavior of many receptors, channels and transporters (Chesler, 1990), including NMDARs, which are inhibited by H^+^ around physiological pH (Tang et al., 1990). In the ischemic brain, tissue and extracellular pH typically drops within minutes toward 6.5 or lower, due to reliance on anerobic glycolysis for ATP resynthesis (Ljunggren et al., 1974; Kraig et al., 1985; Dennis et al., 1991; Katsura et al., 1991), an increase in extracellular H^+^ sufficient to attenuate NMDAR channel openings (Tang et al., 1990) and NOX2 activity (see above). Expectedly NMDAR-mediated excitotoxicity is also reduced (Giffard et al., 1990b; Tombaugh and Sapolsky, 1990).

Despite reducing NMDAR and NOX activation, acidosis is still likely a net contributor to ischemic brain damage as long postulated, as movements of H^+^ or H^+^ equivalents amplify excitotoxicity. Ischemic acidosis enhances neurotoxic Ca^2+^ overload via the gating of acid-sensing ion channels (ASICs – see below). Furthermore, several studies have shown that glutamate- or NMDA-induced Ca^2+^ influx into hippocampal neurons is accompanied by rapid intracellular acidification, in part due to Ca^2+^ displacement of H^+^ from intracellular binding sites (Hartley and Dubinsky, 1993; Irwin et al., 1994; Koch and Barish, 1994). NOX2 activity (Lam et al., 2013) and electroneutral operation of the plasma membrane calcium ATPase (PMCA) may also contribute, the latter importing 2 H^+^ ions for every Ca^2+^ ion exported (Stawarski et al., 2020). Intracellular pH dropped initially to around 6.5 during excitotoxic glutamate exposure (500 μM × 5 m) before progressively recovering and overshooting to pH 7.5–8.0 as a result of acid-extrusion mechanisms, especially membrane Na^+^/H^+^ exchangers (NHEs) (Raley-Susman et al., 1991; Rathje et al., 2013). Sustained operation of NHEs loads intracellular Na^+^, which then raises intracellular Ca^2+^ via NCXs. Consistent with this sequence contributing to excitotoxicity, inhibition of NHE-1 with cariporide reduced glutamate-induced cortical neuronal death, as well as three predicted intermediate steps: a later increase in [Ca^2+^]_*i*_, loss of mitochondrial membrane potential, and intracellular production of ROS (Lee and Jung, 2012). *NHE*^–/–^ mice exhibited reduced infarction after tMCAO (Luo et al., 2005).

In addition, ischemic acidosis is likely itself cytotoxic. Exposure to pH 6.5 for 6 h is lethal to both neurons and glia (Nedergaard et al., 1991). Astrocytes are especially vulnerable to proton-induced death (Goldman et al., 1989; Giffard et al., 1990a), and can be killed after only 15–20 m exposure to pH 6.6 if other extracellular ions are altered to levels found in ischemic brain (low Ca^2+^ and Na^+^, high K^+^, hypoxia), conditions likely to impair cellular pH homeostasis (Swanson et al., 1997; Bondarenko and Chesler, 2001). Sustained intracellular acidity likely becomes lethal due to myriad disturbances in protein conformation and essential cellular processes (Nedergaard et al., 1991), including energy failure (Swanson et al., 1997). If cellular H^+^ favors release of ferrous iron (Kraig et al., 1987), the Fenton reaction (Jung et al., 2009) may facilitate hydroxy radical formation, lipid peroxidation, and a “ferroptosis” form of regulated cell death (Xie et al., 2016; Ratan, 2020) if necrosis does not supervene. Proton-induced death of cerebellar neurons is accompanied by an increase in [Zn^2+^]_*i*_ and reduced by Zn^2+^ chelation, raising the possibility that disturbance in Zn^2+^ storage or other homeostatic mechanisms may also contribute to H^+^ cytotoxicity (Isaev et al., 2010).

### Potassium

A core property of the cation channels gated by ionotropic glutamate receptors, besides permeability to Na^+^, and in certain cases Ca^2+^/Zn^2+^, is high permeability to K^+^ (Nowak et al., 1984). Although glutamate receptor-mediated K^+^ movement has received less attention than Na^+^ or Ca^2+^ movement, it may contribute significantly to excitotoxic apoptosis after mild insults or under other conditions where excitotoxic necrosis is blunted.

Noting that the hallmark cell volume loss occurring during apoptosis would require K^+^ to exit, my laboratory examined K^+^ currents in cortical neurons undergoing serum deprivation-induced apoptosis and observed early enhancement of the delayed rectifier current, *I*_*K*_. Attenuating this current with tetraethylammonium (TEA) or raising extracellular K^+^ inhibited apoptosis, even if [Ca^2+^]_*i*_ was kept at resting levels with gadolinium (Yu et al., 1997). This implication of K^+^ efflux in neuronal apoptosis converged with observations implicating that efflux in other cell types (Bortner and Cidlowski, 2007), and in particular two findings: 1) lymphocyte apoptosis required enough K^+^ efflux to lower [K^+^]_*i*_ to less than 50 mM (Bortner et al., 1997); and 2) in those cells, activation of pro-caspase-3 by cytochrome c and apoptotic nuclease activity was [K^+^]_*i*_ -dependent, inhibited by normal [K^+^]_*i*_ and released by lowered [K^+^]_*i*_ (Hughes et al., 1997).

Supporting the notion that K^+^ efflux may promote apoptosis after mild excitotoxic insults, in a cortical culture model of NMDA-induced neuronal apoptosis (achieved by using a low concentration of NMDA, lowering extracellular Na^+^ and Ca^2+^ to levels found in the ischemic brain and extending exposure time), NMDAR activation markedly reduced cellular K^+^ content (Yu et al., 1999). Furthermore, intraventricular injection of TEA reduced infarction in rats after tMCAO, a finding contrary to prediction from a classic excitation / excitotoxicity standpoint, but consistent with participation of K^+^ efflux in ischemic apoptosis (Wei et al., 2003).

### Zinc

Yet another less-studied cation that participates in excitotoxicity is zinc (Choi and Koh, 1998; Frederickson et al., 2004; Sensi and Jeng, 2004; Granzotto and Sensi, 2015). Zn^2+^ is a dietary requirement and has many essential roles in cell biology (Parisi and Vallee, 1969; Maret, 2017), including service as a central neurotransmitter/neuromodulator regulating circuit behavior and synaptic plasticity (Frederickson, 1989; Smart et al., 1994; Frederickson et al., 2005). Reactive Zn^2+^ is stored in presynaptic vesicles within a subset of glutamatergic nerve terminals throughout CNS and co-released with glutamate in a Ca^2+^-dependent fashion upon nerve terminal firing. Synaptically released Zn^2+^ modifies the behavior of multiple moieties on postsynaptic membranes, including voltage- and agonist-gated channels, with primary signaling actions likely on NMDA and AMPA receptors (Peters et al., 1987; Westbrook and Mayer, 1987; Kalappa et al., 2015). It can also enter (and likely leave) postsynaptic target neurons though Ca^2+^ routes that might equally well be called Ca^2+^/ Zn^2+^ routes, including voltage-gated Ca^2+^ channels, NMDARs, Ca^2+^-permeable AMPARs, TRP channels, ASIC1a and in depolarized cells, reverse operation of NCXs (Sensi et al., 1997; Kerchner et al., 2000; Inoue et al., 2015), the last augmented by intracellular H^+^ and NHE operation (Kang et al., 2020). Ischemic acidosis may also favor Zn^2+^ entry over Ca^2+^ entry through voltage-gated Ca^2+^ channels (Sheline et al., 2002). Gating of unselective Panx1 channels, on neurons by NMDAR-Src signaling, and on astrocytes and oligodendrocytes by P2X7 signaling, might be another route for cellular Zn^2+^ entry or egress (see below).

Once “translocated” from afferent terminals into postsynaptic target cells, Zn^2+^ participates in myriad intracellular signaling pathways including the activation of a metabotropic Zn^2+^ receptor, mZnR/GPR39, and inducing intracellular Ca^2+^ release (Besser et al., 2009). Additional to translocated Zn^2+^, a major source of intracellular signaling Zn^2+^ is mobilization from intracellular stores, especially metallothioneins, by oxidation reactions (Maret, 1994).

Cellular [Zn^2+^]_*i*_ is normally tightly regulated, but in ischemia this homeostasis is disrupted. Release from depolarized nerve terminals and oxidized intracellular stores, together with failure of energy dependent transport, produces increases in extracellular and intracellular Zn^2+^ that can be cytotoxic, especially to neurons (Yokoyama et al., 1986; Lobner et al., 2000; Bozym et al., 2010). Zn^2+^ like glutamate induces apoptosis at lower toxic exposures and necrosis at higher exposures (Manev et al., 1997; Lobner et al., 2000). The parallel between glutamate and Zn^2+^ neurotransmission (Frederickson, 1989) thus extends to excitotoxicity: both are potential transmitter-killers in the ischemic brain.

Evidence that Zn^2+^ dysregulation contributes to acute brain injury was provided by observations of apparent Zn^2+^ translocation from afferent terminals to the cytoplasm of neurons degenerating after sustained seizures or transient global ischemia (Sloviter, 1985; Tønder et al., 1990), and supported by the finding that intraventricular CaEDTA, which chelates extracellular Zn^2+^, blocked both cytoplasmic Zn^2+^ accumulation and degenerative changes in vulnerable neurons after TGI (Koh et al., 1996). At the time we thought CaEDTA was only chelating Zn^2+^ released from nerve terminals, but later studies indicated that CaEDTA can also pull Zn^2+^ from intracellular stores (Frederickson et al., 2002; Lavoie et al., 2007). Calderone et al. (2004) determined that the major accumulation of Zn^2+^ in vulnerable hippocampal CA1 neurons occurred more than 48 h after TGI, and that early injection of CaEDTA attenuated later appearance of Ca^2+^-permeable AMPARs, consistent with the possibility that loss of cellular Zn^2+^ homeostasis constituted a trigger for reducing GluR2 expression. Contribution of Zn^2+^ to infarction after short duration tMCAO was similarly detected: intraventricular CaEDTA reduced infarct volume 3 days later, but this protective effect was lost after full maturation of the infarct 11 days later, suggesting that an early Zn^2+^-triggered component of ischemic infarction was eventually overtaken by other injuries (Lee et al., 2002).

Zn^2+^ like Ca^2+^ also serves as a downstream mediator of excitotoxicity – the latter’s shadow, unleashed and dysregulated by glutamate receptor overactivation. The relationship between Zn^2+^ and Ca^2+^ in mediating excitotoxicity is complex, with target overlap and reciprocal influences; and delineating roles has been further challenged by lack of specificity in historical Ca^2+^ assay tools such as fura-2 (Cheng and Reynolds, 1998). Studies with Zn^2+^ selective dyes indicated that OGD induced an early increase in [Zn^2+^]_*i*_ within hippocampal slice CA1 neurons; a Zn^2+^ chelator blocked this increase and attenuated neuronal death (Stork and Li, 2006). Further examination revealed that an OGD-induced increase in neuronal [Zn^2+^]_*i*_ preceded mitochondrial depolarization, Ca^2+^ deregulation and membrane failure, with Zn^2+^ likely entering mitochondria and contributing to loss of mitochondrial membrane potential (Medvedeva et al., 2009). This meshed with earlier demonstration that elevated intracellular Zn^2+^, like Ca^2+^, can enter and damage mitochondria, leading to their swelling, loss of membrane potential and, at high levels of Zn^2+^, increased ROS generation (Sensi et al., 2003; Clausen et al., 2013), the last likely a consequence of disturbances in mitochondrial electron transport as well as Zn^2+^ induction of p47^*PHOX*^ and increased NOX activity (Noh and Koh, 2000; Slepchenko et al., 2017). Downstream ROS/RNS-induced release of Zn^2+^ from intracellular stores (Berendji et al., 1997; Cuajungco and Lees, 1998; Aizenman et al., 2000) thus drives further oxidative stress. Other injury-promoting events linked to Zn^2+^ overload include activation of nNOS, PARP (Kim and Koh, 2002) and cyclin-dependent kinase 5 (Cdk5 – Tuo et al., 2018; see Wang et al., 2003; Ko et al., 2019) for links to excitotoxicity). Neuronal Zn^2+^ overload together with Ca^2+^-activated CaMKII increase insertion of delayed rectifier Kv2.1 channels into the plasma membrane, increasing K^+^ efflux and facilitating apoptosis (Aras et al., 2009; McCord and Aizenman, 2013). Zn^2+^ may also upregulate NMDAR activity through a Src-dependent mechanism (Manzerra et al., 2001).

In addition to promoting neuronal death, excitotoxic Zn^2+^ dysregulation may contribute to the death of adjacent non-neuronal cells in the ischemic brain. Zn^2+^-induced death of astrocytes was potentiated by concurrent hypoxia and reduced by PARP knockdown (Pan et al., 2013). Intracellular Zn^2+^ release was implicated in mediating peroxynitrite-induced death of oligodendrocytes, through activation of ERK42/44, 12-lipoxygenase and further ROS generation, rather than immediately through hydroxyl radical formation (Zhang et al., 2006). OGD-induced death of cultured oligodendrocytes was attenuated by Zn^2+^ chelation or PARP inhibition (Domercq et al., 2013). Hydrogen peroxide-induced death of endothelial cells (Wiseman et al., 2007) and astrocytes (Furuta et al., 2019) was also attenuated by Zn^2+^ chelators At sublethal levels, Zn^2+^ can upregulate ICAM-1 expression in vascular endothelial cells, thereby promoting leukocyte attraction and microvascular leakage (Sumagin et al., 2008; Yeh et al., 2011).

Together, these observations on glia and endothelial cells suggest that reduction of downstream Zn^2+^ toxicity may help account for the observed ability of glutamate antagonists to reduce brain infarction, not just neuronal death, in animal models of stroke.

### Other Cation Channels

Several other membrane channels, likely activated in part consequential to glutamate receptor overstimulation, can contribute to toxic Ca^2+^/ Zn^2+^ overload and other ionic derangements in the ischemic brain:

1. Transient receptor potential channels. These are variably selective cation channels, subdivided into 7 groups and including receptor-, second messenger- and store-operated members (Venkatachalam and Montell, 2007). Two members of the melastatin group, TRPM7 and TRPM2 are plasma membrane channels highly expressed in brain and implicated in the pathogenesis of ischemic brain damage. TRPM7 is activated in the ischemic brain by ROS/RNS and augments Ca^2+^ overload and oxidative stress (Aarts et al., 2003). Suppressing TRPM7 expression in cortical cultures subjected to either OGD or blocked OGD attenuated Ca^2+^ uptake and neuronal death, leading Aarts et al. (2003) to suggest that TRPM7 might be a foreground pathway for neuronal Ca^2+^ influx into anoxic neurons. Notably, the divalent cation most permeable through TRPM7 is Zn^2+^ (Monteilh-Zoller et al., 2003).
TRPM2 is opened by ADP-ribose accumulating secondary to PARP activity downstream of oxidative DNA damage (Kaneko et al., 2006; Li et al., 2015). Knockdown of TRPM2 expression or drug inhibitors protected male but not female cortical neurons in culture from OGD-induced death, and similarly reduced infarction after focal ischemia in male but not female mice (Jia et al., 2011).
2. Acid-sensing ion channels are part of the Epithelial Na channel/degenerin (ENaC/DEG) family of monovalent plasma membrane cation channels widely expressed throughout the nervous system and gated by extracellular protons (Vullo and Kellenberger, 2020). Xiong et al. (2004) found that pH 6.0 activation of ASIC1a, which is permeable to divalent cations, mediated Ca^2+^-dependent neuronal injury and augmented the neuronal death induced in cortical cultures by blocked OGD. The ability of ischemic acidosis to activate ASICs is potentiated by arachidonic acid liberated by phospholipase A_2_ (Allen and Attwell, 2002), as well as by NO, extracellular Zn^2+^, and Ca^2+^ signaling through CaMKII (Gao et al., 2004; Leng et al., 2014). Mice lacking ASIC1a or pretreated with blocking PcTX venom developed smaller infarcts after tMCAO, and the protective effect of PcTX added to that of the NMDA antagonist, memantine (see below). In a combined pH 6.0 + blocked OGD paradigm, reducing TRP7 activation by adding an antioxidant or NOS inhibitor was not neuroprotective (Xiong et al., 2004) – perhaps another example of one pathway masking another in a parallel race to death.
3. Pannexin ion channels constitute 3 new members of the gap junction superfamily, first cloned from mammalian tissue 20 years ago (Barbe et al., 2006; Yeung et al., 2020). Panx1 is widely expressed on the plasma membrane of central neurons, glia, and endothelial cells. While constitutively permeable to Cl^–^, when activated Panx1 becomes a large conductance pore unspecifically permeable to ions as well as to some larger molecules such as ATP and dyes. It can be activated downstream of NMDARs, primarily through a Src kinase signalosome, but possibly additionally through Ca^2+^ or NO signaling (Zhang et al., 2008; Weilinger et al., 2012). Panx1 can also be activated by P2X7 purinergic receptors and irreversibly activated by C-terminal cleavage via caspases, an event that likely contributes to the execution of apoptosis.
NMDAR-Src activation of Panx1 contributes heavily to anoxic depolarization (Thompson et al., 2006; Weilinger et al., 2012). This linkage positions Panx1 as an excitotoxicity amplifier, operating in parallel with the NMDA channel to augment disturbances in Ca^2+^, Zn^2+^, Na^+^, and K^+^. Administration of a novel peptide interfering with NMDAR-Src activation of Panx1, TAT-Panx_308_, reduced OGD-induced elevation in neuronal [Ca^2+^]_*i*_, mitochondrial membrane permeabilization and neuronal death in hippocampal cultures, as well as infarction after tMCAO in rats (Weilinger et al., 2016).
4. Store-operated Ca^2+^ entry provides Ca^2+^ entry and replenishment of ER Ca^2+^ stores in both non-excitable cells and excitable cells, and has been implicated in the pathogenesis of excitotoxicity and ischemic brain damage (Li et al., 2015; Serwach and Gruszczynska-Biegala, 2019). The system consists of Orai1-3, plasma membrane channels selective for Ca^2+^ [blocked by Zn^2+^ (Gore et al., 2004)] and gated by the ER Ca^2+^ sensors, stomal interaction molecule isoforms, STIM1-2. Release of ER Ca^2+^ induces STIM oligomerization and migration to ER-plasma membrane junctions, where these open Orai to permit cytoplasmic Ca^2+^ entry, which is then taken up by ER via SERCA ATP pumps. As noted above, Group I mGluR activation likely contributes to excitotoxic Ca^2+^ overload by triggering ER Ca^2+^ release, placing SOCE in an enabling role. STIM2 is prominently expressed in brain, and *stim2*^–/–^ mice exhibited reduced neuronal vulnerability to hypoxia *in vitro* and infarction after tMCAO *in vivo* (Berna-Erro et al., 2009).

### Excitotoxic Glial Cell Death

Although excitotoxicity was originally described as specific to neurons, oligodendrocytes express a full array of glutamate receptors (Káradóttir and Attwell, 2007) and are highly vulnerable to excitotoxic injury and death. Young cultured oligodendrocytes can be killed by 24 h exposure to kainate or glutamate; this toxicity was blocked by an AMPAR/KAR antagonist and enhanced by cyclothiazide, which reduces AMPAR desensitization (Yoshioka et al., 1995). Vulnerability to a non-glutamate receptor-mediated mechanism involving inhibition of cystine update by high concentrations of glutamate was also demonstrated (Murphy et al., 1989; Oka et al., 1993). More prominent AMPAR/KAR-mediated, extracellular Ca^2+^-dependent excitotoxicity was demonstrated on cultured optic nerve oligodendrocytes, and a 5–7 days infusion of kainate destroyed optic nerve oligodendrocytes *in vivo* (Matute et al., 1997). Similarly, after maturing 3–5 weeks on a monolayer of astrocytes, oligodendrocyte vulnerability to AMPAR/KAR-mediated excitotoxicity was comparable to that of neurons, with death induced by exposure to as little as 30 μM AMPA for 3 h (McDonald et al., 1998). OGD triggered AMPAR/KAR-mediated oligodendrocyte death in cultures (McDonald et al., 1998) or in adult brain slices (Tekkök and Goldberg, 2001). Ca^2+^ -permeable AMPA receptors are expressed on oligodendrocytes (Matute et al., 1997; Barron and Kim, 2019) and may substantially mediate this excitotoxic vulnerability. Loss of cellular Zn^2+^ homeostasis may contribute, as oligodendrocytes exposed to OGD developed increased [Zn^2+^]_*i*_ and subsequent death was reduced by a Zn^2+^ chelator (Domercq et al., 2013).

In the presence of AMPAR/KAR blockade, OGD induced extracellular Ca^2+^-dependent damage to oligodendrocyte myelinating processes (Salter and Fern, 2005), likely mediated by process-specific expression of an unusual NR3-containing NMDAR relatively insensitive to Mg^2+^ block (Káradóttir et al., 2005). Another contributor to ischemic oligodendrocyte damage may be the their Panx1 channels, possibly activated by P2X7 receptors and ATP released from nearby dying or permeabilized cells (Domercq et al., 2010).

Most astrocytes express AMPARs and mGluRs, but NMDARs and Ca^2+^-permeable AMPARs are generally not abundant (Bradley and Challiss, 2012; Ceprian and Fulton, 2019; Skowrońska et al., 2019). In contrast to oligodendrocytes, astrocytes appear to be relatively insensitive to excitotoxicity, although as noted above they are vulnerable to Zn^2+^ or H^+^-induced damage, and they express Panx1 channels (Iglesias et al., 2009). Hence their death could be enhanced secondary to excitotoxicity occurring in nearby neurons or oligodendrocytes.

## Blocking Excitotoxicity in the Ischemic Human Brain

It is time to get back in the water.

Failure of the first wave of NMDA antagonists in stroke trials, viewed with the easy clarity of informed hindsight, does not come close to excluding excitotoxicity as a major driver of ischemic brain damage or the feasibility of reducing that damage with anti-excitotoxic approaches. Those drugs needed to be in the brain at sufficient levels to ameliorate excitotoxicity at stroke onset, not > 3 h later when efficacy can no longer be demonstrated in animal models. Patients with slowly progressive vascular occlusions might still have benefited, but in many patients the drugs would have entered brain after cells were largely en route to death in a glutamate receptor-independent fashion. Neurons in the outer stroke penumbra with less severe NMDAR overactivation would have been the slowest to die, but there pan NMDA receptor blockade may have sometimes lowered [Ca^2+^]_*i*_ too far and exacerbated regulated cell deaths (Fricker et al., 2018).

Over the last 25 years much has been added to the basic NMDAR-Ca^2+^ overload scenario that served as the basis for initial NMDA antagonist/stroke trials. Returning to an earlier process framework for excitotoxicity (induction, amplification, expression - (Choi, 1992), I would highlight the following events in an updated four-stage working scenario:

1. Induction. Excitotoxicity in the ischemic brain is predominantly triggered by overactivation of neuronal NR2B receptors at stroke onset, and in the ischemic penumbra later, raising [Ca^2+^]_*i*_, activating nNOS and NOX2, and generating ROS/RNS. Concurrent overactivation of other NMDARs, AMPARs, KARs, and mGluR1 augments neuronal Ca^2+^ overload. Ca^2+^-permeable AMPARs, KARs, and NR3-containing NMDARs mediate Ca^2+^ overload in oligodendrocytes; reverse operation of NCXs convert Na^+^ entry to Ca^2+^ overload in axons. Zn^2+^ overload shadows Ca^2+^ overload everywhere. Intracellular pH falls and intracellular K^+^ begins to leave.
2. Amplification. Excitotoxic Ca^2+^/Zn^2+^ overload is promoted by the activation of other plasmalemmal channels, including TRPM7, TRPM2, ASIC1, Panx1 and Orai; NCXs enhanced by Na^+^ import via NHEs; oxidative Zn^2+^ release from intracellular metallothioneins; and in some neurons, increasing expression of Ca^2+^/Zn^2+^-permeable AMPARs. Astrocytic swelling and oxidative stress activate the volume-regulated anion channel (VRAC – see below), facilitating glutamate release and intensifying excitotoxic induction.
3. Expression. An expanding array of concurrent injury and signaling pathways, activated by Ca^2+^/Zn^2+^ overload, K^+^ efflux, intracellular H^+^ and oxidative stress head toward regulated cell deaths over the next hours to days. DNA damage and PARP activation, membrane ATPases, and mitochondrial failure deplete energy stores. Catabolic enzymes, especially phospholipase A_2_ and calpains (Markgraf et al., 1998), damage cellular structures and promote ROS/RNS formation. Increasing intracellular H^+^ synergizes with elevated [Zn^2+^]_*i*_ to damage all cells. In severely ischemic cells (core, inner penumbra), fulminant energy and structural failure culminate in necrosis before a regulated cell death can complete. Oxidative damage is enhanced if reperfusion occurs (Traystman et al., 1991), which it does with increasing frequency in human stroke today due to interventions.
4. Later events. Excitotoxicity *per se* triggers and augments subsequent inflammatory processes to continue destroying brain tissue. The neurovascular unit (neurons, glia, vascular elements) releases cytokines and chemokines, recruiting leukocytes to the evolving infarct over hours to days, advancing microvascular damage and oxidative stress (Zoppo et al., 2000; Anrather and Iadecola, 2016; Jayaraj et al., 2019). In particular, iNOS is expressed in infiltrating neutrophils and endothelial cells 6–96 h after MCAO in rats and in human cerebral infarcts (Forster et al., 1999), adding NO fuel to the fire and synergizing oxidatively with superoxide emanating from neutrophil NOX2 (Tang et al., 2011) and endothelial NOX4. Remarkably, inhibition of iNOS with aminoguanidine reduced infarct volume after pMCAO in rats when given 24 h later (Iadecola et al., 1995). Cellular release of lysosomal cathepsin B may also contribute to remote cell damage and later secondary degeneration (Zuo et al., 2018).

There have also been significant advances in relevant drug development methodologies and clinical trial capabilities. In the wake of the first NMDA antagonist failures, academic and industry investigators met to devise guidelines for improving the testing of candidate neuroprotective treatments in animal models and human trials (Stroke Therapy Academic Industry Roundtable, 1999; Fisher et al., 2009). Vitally, in the interim stroke-is-untreatable nihilism has departed from medicine, as both medical (tissue plasminogen activator, tPA) thrombolysis and, more recently, endovascular thrombectomy improve outcomes in selected patients, even if the latter is delayed up to 24 h after stroke onset (Malik et al., 2020). Major hospitals now deploy specialized stroke teams, capable of completing brain imaging and delivering drugs within minutes of a patient’s arrival, and emergency medical services are tuned to getting patients to stroke centers as quickly as possible. In some communities, mobile stroke vans can image and treat patients in remote locations.

Neuroprotective treatment aimed at reducing ischemic excitotoxicity could be in theory directed at points anywhere along the causality chain from excessive extracellular accumulation of glutamate to the downstream activation of regulated cell death pathways and destruction of cellular structures. Upstream targeting has the general advantage of gaining leverage on multiple divergent downstream pathways but requires early intervention and could be problematic in terms of side effects or interference with beneficial downstream events. Downstream targeting has the advantage of a potentially longer therapeutic time window, greater specificity and less side effects, but risks lower efficacy if unblocked pathways still reach cell death.

Safely reducing glutamate release from depolarized/de-energized neurons seems a stretch goal, although observations noted above with a GluK1R antagonist are worth following up, and reverse operation of neuronal transporters has been proposed to be a major mediator (Rossi et al., 2000). Ischemic glutamate release from astrocytes may be a more promising target, as this appears substantially mediated by druggable volume-regulated anion channels (VRACs). VRACs are activated by cell swelling and oxidative stress, and permeable to certain organic anions including glutamate (Haskew-Layton et al., 2005). Astrocyte-specific deletion of the obligate VRAC subunit gene, *Swell1*, reduced brain infarction in mice subjected to tMCAO (Yang et al., 2019). This excitotoxicity amplification mechanism might be specifically targeted by VRAC inhibitors, or unspecifically and collaterally targeted by antioxidant approaches (Kimelberg, 2005; Dohare et al., 2014).

Blocking all glutamate receptors at stroke onset would likely have great anti-excitotoxic efficacy even against initial excitotoxic necrosis, but widespread loss of fast synaptic excitation would likely be poorly tolerated. Pan block of NMDARs could be tried again, achieved with a short-acting drug that could be withdrawn in time to avoid later Ca^2+^ starvation. However, as cells in an evolving stroke are unlikely to be synchronously in the same state, getting this timing right might be challenging. Future advances in methods for staging the progression of stroke pathophysiology or for location-specific drug delivery might bring this approach back into active consideration.

Block of NR2B is an appealing upstream approach, especially given experience suggesting that this is doable with an acceptable level of side effects. If the strong Ca^2+^ source specificity hypothesis turns out correct, then full block should be the goal to interdict all death signaling. The Ca^2+^ setpoint perspective (and the complication of triheteromeric NR2A + NR2B receptors) would favor a more conservative goal of partial block with a low affinity antagonist that would spare some potentially anti-apoptotic Ca^2+^ signaling. A South Korean biotechnology company, GNT Pharma Co., has developed such a new molecular entity NR2B antagonist, Neu2000 / Nelonemdaz, which also has potent antioxidant properties (Cho et al., 2010; Visavadiya et al., 2013). Nelonemdaz has a favorable profile in animal stroke model paradigms including delayed treatment of tMCAO and pMCAO, and is currently in phase II clinical trials in patients undergoing endovascular thrombectomy (SONIC) (Hong et al., 2018) or presenting with acute ischemic stroke (ENIS I). Another way to improve the therapeutic index of NR2B antagonists may be to dial in pH sensitivity, so that greater block would occur in brain regions with severe ischemia and low pH, and lesser block in brain regions with more normal pH (Yuan et al., 2015).

Moving one step downstream, one might seek to decouple NR2B from signalosome interactions mediating toxicity, including PSD-95-nNOS, Src-Panx1 and possibly PI3K-NOX2, while leaving NR2B channel gating intact. Tymianski and colleagues have explored this latter approach, using a novel eicosapeptide, Tat-NR2B9c that disrupts the interaction of the NR2B C-terminal region with PSD-95 (Ballarin and Tymianski, 2018). Tat-NR2B9c did not block NMDAR-mediated Ca^2+^ influx, but reduced NMDA-induced neuronal death even if applied 1 h after NMDA (Aarts et al., 2002), and also reduced infarction when applied 3 h after tMCAO or 1 h after pMCAO (Sun et al., 2008). Interestingly, another group has proposed that the Tat targeting sequence of Tat-NR2B9c, pulled from a human immunodeficiency virus protein, may have unexpected anti-excitotoxic properties independent of its NR2B9c payload, perhaps mediated by inducing endocytic internalization of membrane ion channels (Meloni et al., 2015).

A Toronto-based biotech company, NoNO Inc. has taken Tat-NR9B2c (NA-1 / Nerinetide) into clinical development. In a phase II trial (ENACT), 185 patients undergoing endovascular repair of intracranial aneurysms received NA-1 or placebo at the end of their procedure, and the NA-1 treated group sustained fewer ischemic infarcts (Hill et al., 2012). The results of a just-completed phase III trial of Nerinetide (ESCAPE-NA1) in 1105 patients with acute ischemic stroke were less encouraging, with no difference in clinical outcome 3 m after stroke. However an exploratory analysis suggested that drug treatment within a 12 h window was associated with improved outcome in the subset of patients that did not receive tPA (Hill et al., 2020). The investigators have hypothesized that plasmin generated by tPA may have cleaved Nerinetide.

Inhibiting many individual steps implicated in augmenting excitotoxic induction (e.g., blocking mGluR1, AMPAR, or KAR activation), or downstream in amplifying or expressing excitotoxicity has been demonstrated to be neuroprotective in animal stroke models, but taking such selective targeting forward into the clinic bears significant risk of being bypassed by concurrent unblocked pathways, especially given the huge variability of human stroke presentations. To achieve a robust protective effect in the human wild, it may prove necessary to inhibit multiple excitotoxic mechanisms. The idea of combination therapies has been endorsed for a long time by many workers in the stroke neuroprotection field. My personal view, after time spent working in the pharmaceutical industry, is that a historical focus on single drug, single mechanism therapies is a central reason why the development of innovative drugs has had a lower success rate in neuroscience than in other areas. Several important drugs, especially in the infectious disease or oncology areas, would disappoint if tested as monotherapies.

An anti-excitotoxic drug combination approach would ideally be configured to ameliorate multiple processes and pathways at both early and late time points. The development and approval process for combination drugs is more complicated than for monotherapies, but not insurmountably so; indeed the number of combination drugs approved by the US FDA has increased each decade since the 1950s, with over 400 combination drugs approved to date (Das et al., 2019). Development of an innovative combination drug can be eased if only one constituent is a new molecular entity and other(s) are previously developed drugs that are well-understood, well-tolerated, and easily available. A growing number of such drugs have useful anti-excitotoxic properties that might boost the efficacy or improve the side effect profile (by permitting lower dosing) of a co-administered new molecular entity anti-excitotoxic therapy. Some examples of candidates for service as a background “partner” (or two) in anti-excitotoxic drug combinations:

1. Memantine. The prototype low-affinity NMDA antagonist, approved since 2003 to treat cognitive deficiency in patients with Alzheimer’s disease and now off-patent. Although NMDA receptor subtype non-selective (Bresink et al., 1996), it may preferentially block extrasynaptic receptors (Xia et al., 2010). It is a low-affinity blocker of the NMDA channel with a fast off-time and use-dependence, properties that likely explain its low side effect profile (Lipton, 2004). It reduces NMDAR-mediated excitotoxicity in cultures and brain infarction after focal ischemia (Chen et al., 2017).
2. Perampanel. Non-competitive AMPA antagonist approved since 2012 as an anticonvulsant. It is still under US patent protection, but this begins to lapse in 2021. The basis for its favorable side effect profile is unclear but perhaps reflects partial antagonism of central AMPA receptors, analogous to memantine on NMDA receptors. It reduces infarction in rats after focal ischemia (Niu et al., 2018), and even if dosed too low to achieve neuroprotection on its own, it might aid the survival of neurons and oligodendrocytes expressing Ca^2+^/Zn^2+^-permeable AMPA receptors.
3. Minocycline. Minocycline is a second generation, brain-penetrant tetracycline antibiotic in clinical use since 1971. Aside from antibiotic properties, it has unexpected neuroprotective effects in a wide range of acute and chronic injury models (Garrido-Mesa et al., 2013), including infarct reduction after focal ischemia in nearly 20 animal studies (Naderi et al., 2019). This ischemic neuroprotection has been largely attributed to inhibition of matrix metalloproteinases (MMPs) at standard antimicrobial doses (Koistinaho et al., 2005). Brain MMP-9 cellular expression and release to the extracellular space is increased after ischemia, likely contributing to inflammation and BBB breakdown (Chaturvedi and Kaczmarek, 2014). However minocycline also inhibits PARP at lower concentrations (10–100 nM) than needed for MMP-9 inhibition (Alano et al., 2006). It has been tested as a monotherapy in human stroke in several small pilot studies, and shown suggestions of benefit (Lampl et al., 2007; Padma Srivastava et al., 2012); a third study was negative, cooling enthusiasm (Kohler et al., 2013).
4. Pyruvate. The glycolytic metabolite, pyruvate, is sold as a human dietary supplement, typically with suggestions that it may aid weight loss or improve athletic performance. High (mM) concentrations of pyruvate reduce Zn^2+^ neurotoxicity *in vitro* (Sheline et al., 2000), a protective effect that may be due to intracellular Zn^2+^ chelation by accumulating citrate and isocitrate (Sul et al., 2016), additional to benefits on cellular NAD^+^ and ATP stores (Sheline et al., 2000). Anti-inflammatory effects have also been described (Wang et al., 2009). Large doses of pyruvate (500 mg/kg) powerfully blocked selective neuronal death in rat hippocampus after TGI (Lee et al., 2001) and 30 m-1 h delayed treatment with lower doses (62.5–125 mg/kg) reduced infarction after tMCAO or pMCAO (Yi et al., 2007). Chronic doses of oral pyruvate (22–44 g/d × 4 weeks) were well tolerated in a human dietary trial, except for some diarrhea (Stanko et al., 1994).
5. Hydrogen. While the physicochemical properties of H_2_ have been long known, recent studies have highlighted how these position H_2_ favorably for use as a biological protectant. H_2_ efficiently quenches toxic hydroxyl and peroxynitrite radicals, but has little effect on the superoxide or hydrogen peroxide molecules that participate in normal cellular signaling (Ohsawa et al., 2007), a profile partially shared with edaravone. H_2_ readily diffuses through tissues and penetrates cell membranes, quenching hydroxyl radicals generated even in cell nuclei. H_2_ exposure is not associated with any known toxicity and hence environmental concerns focus on explosivity (occurring at 41,000 ppm with ambient O_2_). Humans are routinely exposed to H_2_ as a trace gas in air at about 0.5 ppm, and this can reach 7,500 ppm (0.75%) in closed environments such as within submerged submarines (National Research Council, 2008). Inhalation of 49% H_2_ (with 50.2% helium and 0.8% O_2_) in a gas mixture called Hydreliox has been used by human divers.
Administration of H_2_ has been reported to produce protective and anti-inflammatory effects in multiple experimental injury settings, including cardiac ischemia, organ transplantation, sepsis, and 1-methl-4-phenyl-1,2,3,6-tetrahydropyridine (MPTP)-induced dopaminergic neuronal loss (Ohta, 2014). Inhalation of 2% H_2_ gas reduced infarction in rats after tMCAO (Ohsawa et al., 2007) and brain injury in swine after TGI (Cole et al., 2019). Besides radical scavenging, indirect mechanisms, e.g., activation of transcription factor Nrf2, have been suggested to contribute to protective effects (Kawamura et al., 2013). A small randomized controlled trial of H_2_ gas inhalation in stroke patients reported good safety and a hint of benefit (Cole et al., 2019). My colleagues at Stony Brook University have initiated a pilot therapeutic trial of H_2_ in patients with acute ischemic stroke in combination with a 5 days course of minocycline (H2M treatment – ClinicalTrials.gov Identifier NCT03320018).

Another potential partner drug is the endogenous purine metabolite and plasma antioxidant, uric acid (Ames et al., 1981). Uric acid reduces excitotoxicity in culture and infarction after tMCAO in rats (Yu et al., 1998); further reviewed in Llull et al. (2016), and a Phase IIb clinical trial (URICO-ICTUS) examining the effect of a 1 g dose administered together with tPA showed a trend toward better outcomes at 90 days (Chamorro et al., 2014). However uric acid can have pro-oxidant effects in membrane and intracellular compartments, promoting NOX activity and itself forming radicals (Sautin and Johnson, 2008).

Non-pharmacological partner approaches should also be considered. Mild hypothermia attenuates a broad swath of injury cascades, including early and late excitotoxicity pathways, and protects the human brain after TGI (cardiac arrest/resuscitation) (Ginsberg, 2008; Yenari and Han, 2012). Implementation challenges and side effects have slowed efforts to bring hypothermia forward as a monotherapy for stroke, but these issues would recede if it were implemented leniently without need to reach monotherapy effectiveness (Yenari and Han, 2012). As noted by Ginsberg (2008), ischemic brain injury lies on a temperature-dependence continuum: even mild hyperthermia is clearly bad for the injured brain.

There are of course a daunting – factorial, if order matters – number of possible rational drug and dosage combinations. If only a highly optimized combination will succeed, the discovery and development road ahead could end up lengthy, but everything we know at present leaves open the more favorable possibility that many combination approaches now on the table would end up working well enough to be clinically useful. Perhaps Radicava or Nerinetide are already there as monotherapies.

To systematically identify the most promising and robust combinations for clinical testing, the animal efficacy bar might be further raised to the point that most monotherapies would fail. This has been done historically by increasing treatment delay after tMCAO or pMCAO, but it may be that commitment points to various deaths will constitute a hard assay ceiling, limiting ability to differentiate among multiple promising approaches. And a long treatment window *per se* is no longer a requirement for clinical value, as treatment delays are becoming progressively shorter in today’s stroke centers. Other ways to raise the laboratory animal efficacy bar might be to raise core temperature, raise pre-stroke glucose (increasing ischemic acidosis), add concurrent organ stressors, or utilize specific genetic backgrounds prone to severe strokes. Adequate survival times to permit delayed infarction to complete, efficacy in both male and female animals, and demonstration of white matter protection in animal models larger than rodents should also be sought (Stroke Therapy Academic Industry Roundtable, 1999; Fisher et al., 2009).

Stroke remains today a leading cause of death and disability throughout the world (Katan and Luft, 2018), and hypoxic-ischemic CNS injury occurs additionally in other common settings such as head trauma, spinal cord injury, cardiac arrest and surgery. The unabridged strength of the central hypothesis that excitotoxicity damages brain in human stroke, expanded understandings of how excitotoxicity works and where it might be interdicted, readiness of the health care system to manage stroke patients with alacrity (finally), and the accessibility of several promising partner treatments for combination therapies, all suggest that it is time to recharge the global effort to develop anti-excitotoxic neuroprotective drugs for stroke. Unfortunately, in recent years large pharmaceutical companies have substantially shifted investment away from discovering and developing innovative neuroscience drugs, based on return-on-investment assessments (Choi et al., 2014). Alzheimer’s disease R&D may still be on the table, but stroke R&D is certainly not - the failure of earlier stroke neuroprotection trials contributed palpably to corporate de-investment decisions. Thus the recharge will need to be led, at least initially, by academic investigators and biotechnology companies.

## Author Contributions

DC wrote the work and approved it for publication.

## Conflict of Interest

DC is a paid scientific advisor to GNT Pharma Co., Ltd., Seoul, South Korea.
